# Machine learning models for differential diagnosing HER2-low breast cancer: A radiomics approach

**DOI:** 10.1097/MD.0000000000039343

**Published:** 2024-08-16

**Authors:** Xianfei Chen, Minghao Li, Danke Su

**Affiliations:** a Department of Radiology, The First Affiliated Hospital, Hainan Medical University, Haikou, China; b Department of Medical Imaging Center, Guangxi Medical University Cancer Hospital, Nanning, Guangxi, China.

**Keywords:** DCE-MRI, HER2 low expression, HER2-positive breast cancer, machine learning, radiomics

## Abstract

To develop machine learning models based on preoperative dynamic enhanced magnetic resonance imaging (DCE-MRI) radiomics and to explore their potential prognostic value in the differential diagnosis of human epidermal growth factor receptor 2 (HER2)-low from HER2-positive breast cancer (BC). A total of 233 patients with pathologically confirmed invasive breast cancer admitted to our hospital between January 2018 and December 2022 were included in this retrospective analysis. Of these, 103 cases were diagnosed as HER2-positive and 130 cases were HER2 low-expression BC. The Synthetic Minority Oversampling Technique is employed to address the class imbalance problem. Patients were randomly split into a training set (163 cases) and a validation set (70 cases) in a 7:3 ratio. Radiomics features from DCE-MRI second-phase imaging were extracted. Z-score normalization was used to standardize the radiomics features, and Pearson’s correlation coefficient and recursive feature elimination were used to explore the significant features. Prediction models were constructed using 6 machine learning algorithms: logistic regression, random forest, support vector machine, AdaBoost, decision tree, and auto-encoder. Receiver operating characteristic curves were constructed, and predictive models were evaluated according to the area under the curve (AUC), accuracy, sensitivity, and specificity. In the training set, the AUC, accuracy, sensitivity, and specificity of all models were 1.000. However, in the validation set, the auto-encoder model’s AUC, accuracy, sensitivity, and specificity were 0.994, 0.976, 0.972, and 0.978, respectively. The remaining models’ AUC, accuracy, sensitivity, and specificity were 1.000. The DeLong test showed no statistically significant differences between the machine learning models in the training and validation sets (Z = 0, *P* = 1). Our study investigated the feasibility of using DCE-MRI-based radiomics features to predict HER2-low BC. Certain radiomics features showed associations with HER2-low BC and may have predictive value. Machine learning prediction models developed using these radiomics features could be beneficial for distinguishing between HER2-low and HER2-positive BC. These noninvasive preoperative models have the potential to assist in clinical decision-making for HER2-low breast cancer, thereby advancing personalized clinical precision.

## 1. Introduction

Breast cancer (BC) is a heterogeneous disease that manifests clinically in different ways, comprising various subtypes, and demonstrating diverse responses to treatment. It is the primary cause of morbidity and mortality related to cancer in women.^[1,2]^ Approximately 15% to 20% of BC are human epidermal growth factor receptor 2 (HER2)-positive. HER2 receptor tyrosine kinase is encoded by the proto-oncogene HER2, resulting in overexpression of HER2 protein and a highly aggressive tumor phenotype. This leads to a high risk of metastasis and a poor survival rate. However, the introduction of anti-HER2 therapies has significantly advanced HER2-positive BC treatment. Neoadjuvant and adjuvant therapies have improved patient outcomes, particularly in metastatic disease.^[3]^ However, approximately 45% to 55% of patients exhibit low HER2 expression, defined as immunohistochemistry (IHC) 1+ or IHC 2+ without amplifying the HER2 gene in fluorescence in situ hybridization (FISH).^[4,5]^ According to the current guidelines for HER2 validation,^[6]^ these patients were classified as HER2-negative BC. Therefore, available HER2-targeted treatments are not recommended because of their lack of effectiveness. Nevertheless, the development of novel antibody-drug conjugates, such as Trastuzumab Deruxtecan (T-DXd), has enabled patients with HER2-low-expressing BCs to benefit from targeted therapies.^[7]^

Accurate determination and evaluation of different HER2 protein expression levels are crucial for identifying anti-HER2 targeted drugs and selecting the appropriate population. Currently, detecting HER2 status primarily relies on invasive methods such as IHC or FISH, which involve tissue samples.^[8]^ However, there is a lack of agreement in HER2 status between crude needle aspiration biopsies and subsequent excisional biopsies of the same breast cancer, with a range of 81% to 96%.^[9,10]^ Moreover, these techniques are limited by intra-tumor heterogeneity, which is considered a significant factor in cancer treatment failure and poor prognosis.^[11]^ Additionally, the small amount of biopsied tissue may not fully represent the overall characteristics of the tumor. Therefore, noninvasive technologies that can detect HER2 status with high accuracy are urgently required.

Radiomics^[12–14]^ enables the extraction and quantification of subtle imaging characteristics, allowing the exploration of potential relationships between these features and the physiological processes of tumors. Previous research^[12–14]^ has demonstrated that radiomic characteristics derived from dynamic enhanced magnetic resonance imaging (DCE-MRI) are associated with biological traits, tumor heterogeneity, molecular subtypes, Ki-67 expression, and the therapeutic outcome of neoadjuvant chemotherapy. Machine learning (ML),^[15,16]^ an artificial intelligence discipline, uses sophisticated algorithms to identify and analyze inconspicuous lesion characteristics, which may pose challenges to visual interpretation. By acquiring knowledge regarding the unique characteristics of these lesions, ML techniques can support clinicians in making informed decisions and predictions, thereby enhancing the diagnostic accuracy and efficacy of clinical assessments.

Currently, there is limited published research on HER2-low BC.^[17,18]^ We hypothesized that DCE-MRI radiomics is a reliable method for differentiating HER2-low BC. noninvasive detection of HER2-low BC through DCE-MRI radiomics could provide significant benefits for HER2-low patients, especially those with metastatic breast cancer who are not eligible for surgery. This would allow them to receive targeted therapy for antibody-drug conjugates, without requiring multiple biopsies.

To date, there have been no published studies on the accuracy of machine learning models in combination with DCE-MRI-based radiomics for predicting HER2-low breast cancer patients. However, the potential differences in patients with HER2-low breast cancer remain unclear. These uncertainties pose challenges in achieving more precise patient selection and optimizing drug combinations for HER2-low patients in the current era.

Therefore, the main objective of this study was to develop and validate machine learning models based on preoperative DCE-MRI radiomics to differentiate between patients with HER2-low and HER2-positive BC. This endeavor aims to provide a reference and guidance for the clinical advancement of precise and personalized treatment.

## 2. Materials and methods

### 2.1. Clinicopathological information

This retrospective study analyzed 307 patients with invasive breast cancer who underwent DCE-MRI at the Guangxi Medical University Cancer Hospital between January 2018 and December 2022. The patients were confirmed to have invasive breast cancer based on their pathology.

The inclusion criteria for this study were as follows: preoperative DCE-MRI with high-quality images, availability of complete postoperative pathological, IHC, and FISH data, and breast magnetic resonance imaging conducted within 2 weeks before surgery.^[19]^ The exclusion criteria were as follows: patients who had undergone radiotherapy or neoadjuvant chemotherapy before DCE-MRI; patients without DCE-MRI; and patients with HER2-zero expression.

**Figure 1.**
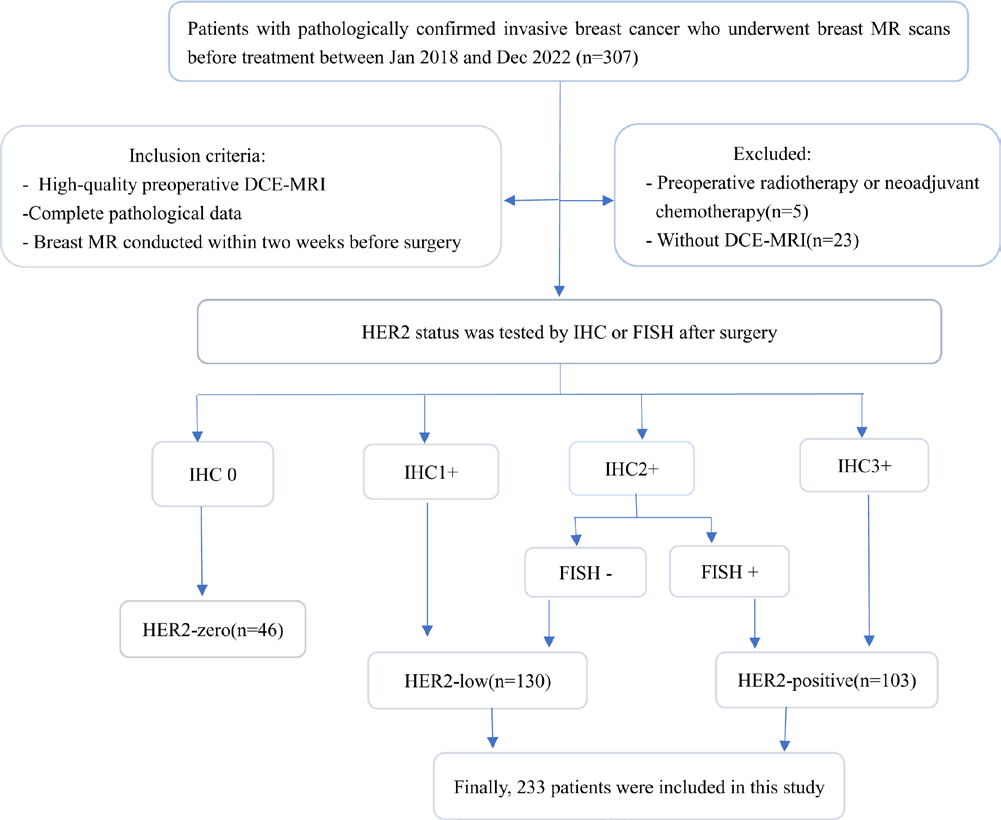
Flowchart of patient recruitment with inclusion and exclusion criteria in this study.

All pathological results were obtained by using an electronic medical record system. The HER2 status of all patients with invasive breast cancer was assessed using IHC, and those with an IHC score of 2 + underwent further evaluation of HER2 status using FISH. Figure 1 the flowchart in this study.

A total of 233 patients were included in the analysis, including 130 patients with HER2 low-expressing (IHC2+, FISH-negative, or IHC1+) and 103 with HER2-positive (IHC3+ or IHC2+, FISH gene amplification). Our institutional review board approved this retrospective investigation conducted in this study and waived the requirement for informed consent (ethics approval number: LW2023079).

### 2.2. Histopathology and immunohistochemistry study

The status of HER2 was determined using IHC or FISH after the operation. Following the guidelines of the American Society of Clinical Oncology/College of American Pathologists (ASCO/CAP),^[6]^ tumors with equivocal scores of 2 + were further tested using FISH. The HER2 results were categorized as HER2-zero (IHC score of 0), HER2-low (IHC score of 1+ or 2+ with negative FISH findings), or HER2-positive (IHC score of 3+ or 2+ with positive FISH results). For the estrogen receptor (ER)/progesterone receptor (PR) examination, tumors were categorized as ER/PR^+^ if nuclear staining was present in ≥1% of the tumor cells.

### 2.3. MR protocols

The study utilized a 3.0-T MRI (GE Discovery 750) system with an eight-channel breast phased-array surface coil to perform the MR scans. Patients were placed in a prone position to ensure proper extension of the mammary glands within the coil. All participants underwent a comprehensive MRI evaluation, which included axial pre-contrast scans with T1-weighted fat saturation, axial sequences with fat suppression for T2-weighted imaging (FS-T2WI), and dynamic contrast-enhanced T1-weighted imaging with fat suppression (DCE-T1WI). The scanning parameters for the unenhanced T1-weighted axial sequences were as follows: repetition time (TR)/echo time (TE) = 640/7.6 ms, field of view (FOV) 320 × 320 mm; matrix, 512 × 512; slice thickness, 4 mm; and slice spacing, 1 mm. For fat-suppressed T2-weighted axial sequences, the parameters were as follows: TR/TE = 2587/85 ms, FOV 320 × 320 mm; matrix, 512 × 512; slice thickness, 4 mm; and slice spacing, 1 mm. The DCE-T1WI was conducted using the volume imaging for breast assessment technique (VIBRANT), with the following parameters: TR/TE = 3.9/1.7 ms, FOV = 360 mm × 360 mm, matrix = 512 × 512, slice thickness = 1 mm, and phase = 8. Before contrast injection, a mask scan was performed, and subsequently, Gd-DTPA was injected via the dorsal vein using a high-pressure syringe at a dose of 0.2 mmol/kg with an injection flow rate of 2.5 mL/s. Following the contrast injection, 25 mL of normal saline was rapidly administered. Subsequently, 8 consecutive scans were performed.

### 2.4. Tumor segmentation and feature extraction

#### 2.4.1. Tumor segmentation

Previous studies have shown that the contrast between the breast cancer mass and background reaches a peak 60 to 120 seconds after contrast injection.^[12]^ In this study, we specifically selected the second phase of DCE-MRI to accurately identify the region of interest (ROI). Because of partial-volume effects on MRI, the boundary of the intratumoral ROI is smaller than that observed in humans.^[12]^ To ensure precise delineation of the tumor boundaries, a radiologist (Read 1 XC) with 15 years of experience in breast imaging manually delineated the ROIs on all slices using ITK-SNAP software (version 3.6.0, http://www.itksnap.org). To minimize bias, radiologists were blinded to the pathological findings. During the segmentation process, careful attention was paid to exclude any areas of necrosis within breast cancer. Subsequently, three-dimensional ROIs of the intratumoral regions were obtained. To assess the stability of the extracted features, another radiologist (Read 2 ML) with 5 years of experience, in addition to Read 1, independently re-extracted the radiomic features from 30 randomly selected patients from the entire study group after 1 month. The intraclass correlation coefficient (ICC) was used to quantify feature consistency and reproducibility. ICC values ranging from 0.5 to 0.75 indicated moderate reliability, values between 0.75 and 0.9 indicated good reliability and values greater than 0.9 demonstrated excellent reliability.^[20]^

#### 2.4.2. Radiomics feature extraction

To ensure consistency and reduce feature variability during radiomics feature extraction, several image pre-processing steps were implemented. These steps include gray discretization, intensity normalization, and voxel resampling.^[21]^ PyRadiomics open-source software (https://github.com/Radiomics/pyradiomics)^[22]^ was used for the feature extraction. All image processing and extracted features adhere to the guidelines set by the Image Biomarker Standardization Initiative (IBSI).^[23]^ Wavelet imaging filters and Laplacian of Gaussian (LoG) filtering were used to process the original images and generate supplementary images to enhance the abundance of features. From the original, LoG and wavelet images, 1046 features were extracted, including 18 first-order statistical features,14 shape-based features, 68 texture features, 688 wavelet features, and 258 LoG features. Among the texture features, there were 22 gray-level co-occurrence matrix (GLCM) features, 16 Gray Level Size zone matrix (GLSZM) features, 16 Gray Level Run Length Matrix (GLRLM) features and 14 Neighboring Gray Level Dependence Matrix (NGLDM) features. These textural features provide valuable insights into tumor heterogeneity. Further details regarding the formulas used to calculate radiomics signatures can be found on the official website (https://pyradiomics.readthedocs.io).

### 2.5. Feature selection and model construction

As previous studies suggested, All patients were randomly divided into a training set (n = 163) and a validation set (n = 70), with proportions of 70% and 30%, respectively.^[12,21,24]^ To address potential bias owing to an unequal distribution of positive and negative samples, we utilized the Synthetic Minority Oversampling Technique^[25]^ resampling method. This technique involves oversampling the minority class (patients with a small number of tumors) strategically and undersampling the majority class (patients with a large number of tumors) randomly, to achieve a balanced distribution of samples across various patient groups.

Radiomic features were standardized using Z-score normalization to ensure a uniform scale of feature values. For dimensionality reduction, Pearson correlation coefficient^[26]^ was used to identify highly correlated feature pairs. If the absolute value of the correlation coefficient was ≥0.99, one of the features was removed. Recursive Feature Elimination^[26,27]^ was then applied to select 10 quantitative features for modeling (more information about recursive feature elimination can be found at https://scikit-learn.org/stable/modules/feature_selection.html#recursive-feature-elimination).

**Figure 2.**
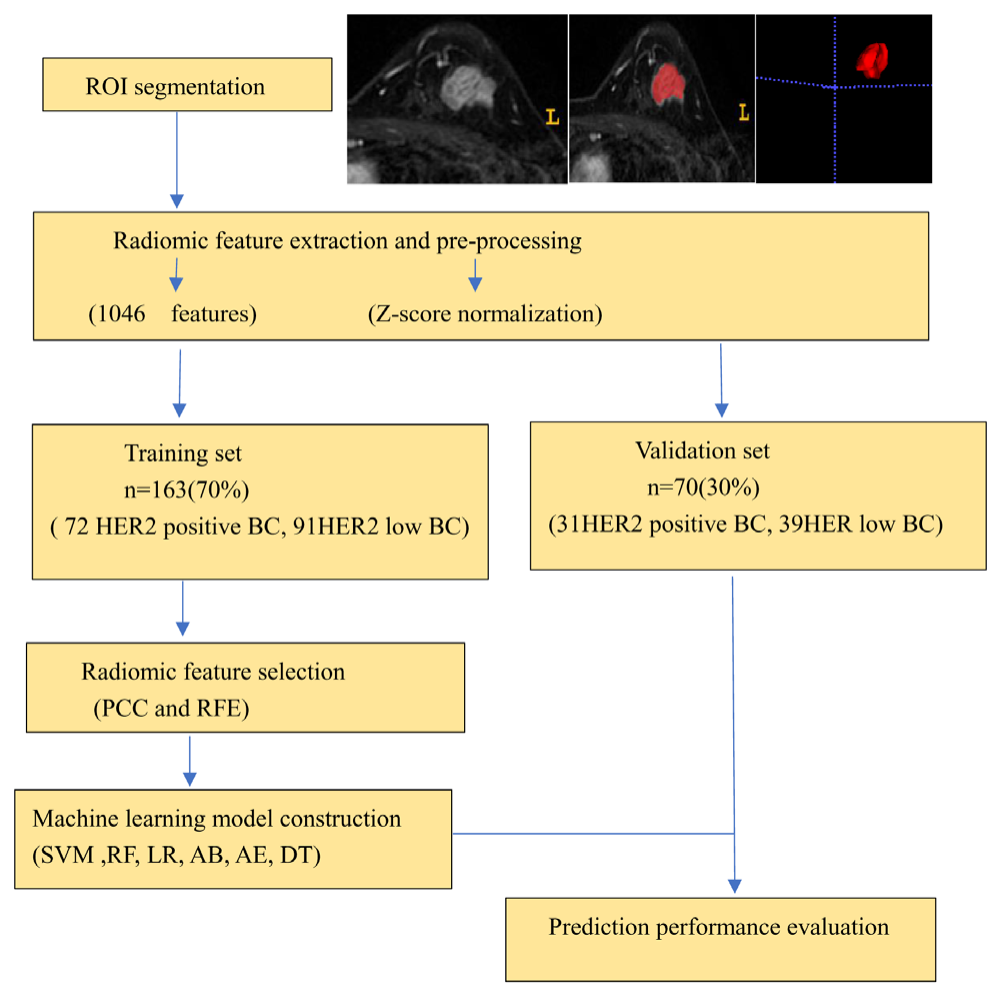
Radiomics workflow. The prediction workflow consisted of 4 main steps: (1) ROI segmentation, (2) radiomic feature extraction and preprocessing, (3) radiomic feature selection, and (4) machine learning model establishment (using a training set), and prediction performance evaluation (using an independent validation set). ROI = the region of interest.

Six supervised classification algorithms – logistic regression (LR), random forest (RF), support vector machine (SVM), AdaBoost (AB), decision tree (DT), and auto-encoder (AE) – were used to construct the prediction models (The details of the machine learning algorithms are described in the Supplementary Material http://links.lww.com/MD/N363 or can be found at https://scikit-learn.org/stable/user_guide.html#). To prevent overfitting, 5-fold cross-validation was implemented.^[28]^ The radiomics workflow is shown in Figure 2.

To assess the performance of these models, receiver operating characteristic curves were constructed and the area under the curve (AUC), accuracy (ACC), specificity (SPE), and sensitivity (SEN) were calculated. The analyses were conducted in Python (version 3.7.6) using the FeAture Explorer Pro (FAE, version 0.5.6). The FAE code can be accessed at https://github.com/salan668/FAE.^[29]^

### 2.6. Statistical analysis

To assess the normality and homogeneity of variance for continuous variables, Shapiro–Wilk and *F* tests were conducted. Continuous variables with normal distributions are presented as mean ± standard deviation (SD) whereas non-normally distributed variables are presented as medians (lower quartile, upper quartile). Categorical data are expressed as relative distribution frequency and percentage. Age comparisons were performed using an independent *t* test, and tumor size was compared using the Mann–Whitney *U* test. The chi-squared test was used for categorical variables. Differences in the area under the curve between the 6 machine learning models were assessed using the DeLong’s test. All statistical analyses were performed using IBM SPSS version 26.0, https://www.ibm.com/spss. Statistical significance was set at *P* < .05.

## 3. Results

### 3.1. Clinicopathologic characteristics

This study included 233 patients with invasive breast cancers. The mean age was 49.92 ± 10.68 (range: 26–83 yr). In the training set, 72 (44.2%) patients were HER2-positive and 91 (55.8%) were HER2-low; in the validation set, 31 (44.3%) were HER2-positive and 39 (55.7%) were HER2-low. PR status differed significantly between the validation and training sets (*P* = .000), whereas no significant differences were found in any other clinicopathological characteristics between the 2 sets (Table 1).

**Table 1:** Clinicopathological characteristics of patients in the training and validation sets.

Characteristics	Training set (n = 163)	Validation set (n = 70)	*Z*/*t*/χ^2^ value	*P* value
Age, years (Mean ± SD)	50.49 ± 10.43	48.60 ± 11.19	1.241	.216
Tumor size on MRI, median (IQR), cm	2.70 (1.90, 4.00)	3.05(2.10, 4.32)	−1.496	.135
Menopausal status				
Pre	93 (57.1%)	42 (60%)	0.174	.676
Post	70 (42.9%)	28 (40%)		
Pathological ALN status			1.976	.192
Positive	91 (55.8%)	46 (65.7%)		
Negative	72 (44.2%)	24 (34.3%)		
ER			3.383	.084
Positive	122 (74.8%)	60 (85.7%)		
Negative	41 (25.2%)	10 (14.3%)		
PR			47.898	.000
Positive	121 (74.2%)	18 (25.7%)		
Negative	42 (25.8%)	52 (74.3%)		
HER2 subgroups			0.000	.987
HER2 -positive	72 (44.2%)	31 (44.3%)		
HER2-low	91 (55.8%)	39 (55.7%)		

ALN = axillary lymph node, ER = estrogen receptor, HER2 = human epidermal growth factor receptor-2, IQR = interquartile range, MRI = magnetic resonance imaging, PR = progesterone receptor, SD = standard deviation.

### 3.2. Intra- and inter-observer agreement for radiomics feature extraction

Ten pertinent features were selected from a pool of 1046 features, encompassing one LoG feature, 8 wavelet features, and one first-order statistical feature. ICC ranged from 0.814 to 0.984, while the inter-observer ICC ranged from 0.840 to 0.974. These results suggest a favorable consistency in feature extraction, both within and between observers (Table 2).

**Table 2:** Intra- and inter-ICC for 10 selected features.

Features	ICC (inter)	ICC (intra)
original_glszm_ZoneEntropy	0.867	0.814
log-sigma-2-0-mm-3D_glszm_SmallAreaLowGrayLevelEmphasis	0.840	0.923
wavelet-LHL_firstorder_MeanAbsoluteDeviation	0.893	0.950
wavelet-LHH_glcm_Imc1	0.940	0.919
wavelet-HLL_firstorder_RobustMeanAbsoluteDeviation	0.956	0.912
wavelet-HLH_firstorder_10Percentile	0.876	0.900
wavelet-HHH_firstorder_10Percentile	0.909	0.949
wavelet-LLL_firstorder_RootMeanSquared	0.896	0.897
wavelet-LLL_glcm_Imc1	0.974	0.984
wavelet-LLL_glrlm_RunVariance	0.892	0.947

ICC (inter) = inter-class correlation coefficient, ICC (intra) = intra-class correlation coefficient.

### 3.3. Prediction performance of machine learning models

**Figure 3.**
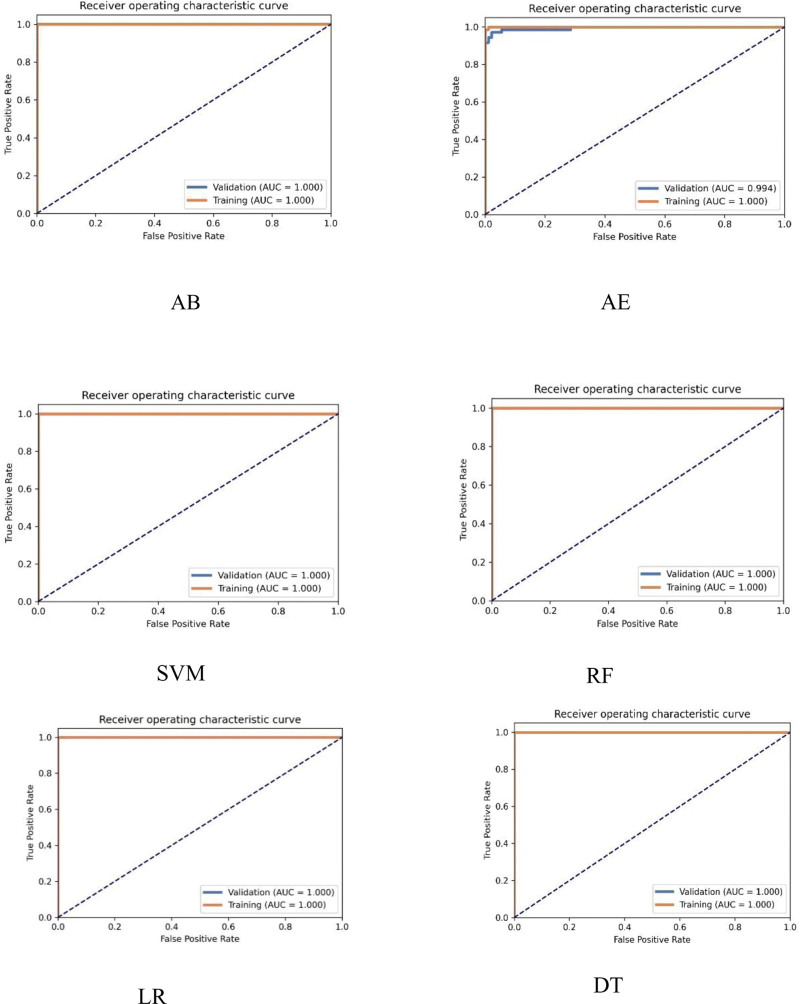
Receiver operating characteristic (ROC) curves of the AB, AE, SVM, RF, LR, and DT models in training and testing cohorts. AB = AdaBoost, AE = auto-encoder, DT = decision tree, LR = logistic regression, RF = random forest, SVM = support vector machine.

Table 3 shows the performance of various ML models. The AUC values for SVM, RF, LR, AB, AE, and DT were all 1.000 in the training set. In the validation set, the AUC of AE was 0.994, whereas the AUC of the other models was 1.000. The DeLong test showed no statistically significant differences between the machine learning models in the training and validation sets (*Z* = 0, *P* = 1). Figure 3 shows the receiver operating characteristic curves of the 6 ML models.

**Table 3:** Prediction performance of the machine learning models.

Machine learning algorithm	Training set				Validation set			
	ACC	SEN	SPE	AUC	ACC	SEN	SPE	AUC
LR	1.000	1.000	1.000	1.000	1.000	1.000	1.000	1.000
SVM	1.000	1.000	1.000	1.000	1.000	1.000	1.000	1.000
RF	1.000	1.000	1.000	1.000	1.000	1.000	1.000	1.000
AB	1.000	1.000	1.000	1.000	1.000	1.000	1.000	1.000
AE	1.000	1.000	1.000	1.000	0.976	0.972	0.978	0.994
DT	1.000	1.000	1.000	1.000	1.000	1.000	1.000	1.000

AB = AdaBoost, ACC = accuracy, AE = auto-encoder, AUC = area under the curve, DT = decision tree, LR = logistic regression, RF = random forest, SEN = sensitivity, SPE = specificity, SVM = support vector machine.

## 4. Discussion

In this retrospective study, we found that certain radiomic features extracted from the second phase of DCE-MRI have satisfactory reproducibility and reliability. Radiomic features derived from DCE-MRI could potentially serve as a noninvasive approach to assess the biological characteristics and heterogeneity of breast cancers. Moreover, the ML model based on radiomics features exhibited outstanding diagnostic performance in distinguishing HER2-low-expressing from HER2-positiveBC.

The distinction between HER2-low expression and HER2-positive invasive breast cancer is crucial for selecting the most appropriate targeted therapy. Currently, HER2 status is primarily determined by IHC or FISH.^[4,5]^ However, these techniques have limitations owing to intratumoral heterogeneity. It is important to note that genetic heterogeneity in BC is a significant factor in treatment failure and poor prognosis.^[2]^ Biopsies that provide limited samples may not fully represent the entire tumor. Radiomics has emerged as an alternative approach for overcoming this limitation. It can convert medical images into high-dimensional data for quantitative analysis.

Previously, HER2-low tumors were not considered suitable for treatment with HER2-directed agents.^[30]^ Recent studies^[31,32]^ have shown that mutations in the PI3K/AKT/mTOR pathway play a significant role in trastuzumab resistance in HER2-low BC patients. In contrast, HER2-positive BC is directly linked to ERBB2 amplification, which is the basis for the effectiveness of conventional anti-HER2 agents in treating these tumors.^[3]^ Differences between these 2 subtypes can be observed at the clinicopathological and molecular levels, with visible macroscopic distinctions on DCE-MRI and subtle detectable variations in high-throughput radiomic signatures.

In this study, we extracted 5 types of features from DCE-MRI: shape-based, first-order statistical, texture-based, wavelet, and LoG features.^[33–35]^ Shape-based features provide quantitative descriptions of the geometric characteristics of the region of interest, such as volume, surface-to-volume ratio, shape, and density, which are essential for assessing tumor characteristics. First-order statistical features consider the distribution of individual voxel values, including mean, median, standard deviation, kurtosis, skewness, energy, entropy, homogeneity, and variance. Texture features describe the statistical relationships between voxels and provide a more comprehensive and detailed understanding of lesions by extracting and quantifying information regarding their regularity, roughness, and grayness, which may not be discernible by visual inspection. Wavelet features^[33]^ are characterized by the localization of spatial frequencies, which can effectively extract the high- and low-frequency signals in the image and analyze the texture changes in a more comprehensive and detailed manner. The Laplacian of Gaussian (LoG),^[36]^ a measure of isotropy of the second-order spatial derivatives of an image, is used to highlight regions of an image where rapid changes in intensity occur and can more plausibly explain spatial variations in tumors. In this study, the 10 most relevant features were screened to construct the model, including one LoG feature, 8 wavelet features, and one first-order statistical feature. Most of these were wavelet features, suggesting that they might be associated with HER2low and HER2positive BC. Similarly, in a study by Zhang et al,^[12]^ wavelet features were extracted to predict molecular subtypes of invasive ductal breast cancer. They concluded that wavelet features provide a substantial amount of detailed information about BC.

ML algorithms exhibit high flexibility and numerous degrees of freedom, potentially leading to overfitting of training data.^[37]^ To address this issue, multiple rounds of training and validation are typically performed to fine-tune the algorithm and prevent overfitting. One widely used technique for this purpose is k-fold cross-validation (CV), where the dataset is randomly divided into k equal-sized subsamples.^[38]^ This approach helps alleviate overfitting by ensuring that the model effectively generalizes to the unseen data. In our study, we implemented 5-fold cross-validation by dividing the dataset into 5 equal parts. The model was trained on 4 parts and evaluated on the remaining part in each iteration, which was repeated 5 times to cover all sections. The overall model performance was computed as the average across all testing sets.^[39]^

ML is a computational approach that leverages data to enhance performance and make precise predictions. In this study, we used 6 supervised classification algorithms: LR, SVM, DT, RF, AE, and AB. These algorithms are commonly utilized in the classification of breast lesions and differentiation of the molecular subtypes of breast cancer.^[39]^ LR models have a fixed number of parameters based on the input features and generate categorical predictions, akin to linear regression fitting points to a line by minimizing a function such as the mean squared error (MSE).^[40]^ SVM functions by transforming data into separate classes using a hyperplane, depicted as a line in a two-dimensional space, with support vectors near the hyperplane playing a crucial role in classification.^[41]^ RF constructs multiple decision trees with varying depths, utilizing random subsets of the dataset and features at each split to create uncorrelated trees that yield an unbiased final predictor.^[41]^ DT is specifically tailored for early breast cancer detection by organizing the dataset into smaller subsets for classification.^[42]^ AB tackles challenges by amalgamating “weak” models to form a diverse and accurate model with robust generalization capabilities.^[40,43]^ An AE, also known as a multilayer perceptron (MLP), is a Neural Network (NN) model used for data classification, linearly combining features, and applying an activation function to theoretically approximate any complex function.^[43]^ This study demonstrates that these 6 supervised classification algorithms, in conjunction with DCE-MR-based radiomics, improve the classification performance in distinguishing between HER2-low and HER2-positive breast cancer patients.

ML algorithms have been used to develop predictive models that combine clinical or imaging data to further improve accuracy.^[16]^ Sheng et al^[44]^ conducted a study on ML-based MRI radiomics combined with clinical features to predict molecular subtypes of invasive breast cancer. The results showed that The RF model performed the best, with an AUC of 0.805, in distinguishing between HER2-positive and non-HER2-positive BCs. Zhou et al^[45]^ utilized an SVM to establish DCE-MRI radiomics-based features for predicting HER2-positive and HER2-negative BCs. The AUC values were 0.71 and 0.68 in the training and validation sets, respectively. Song et al^[46]^ compared 3 algorithms (SVM, LR, and quadratic discriminant analysis) for predicting HER2 2+ and HER2-negative BCs and found that the SVM model outperformed the others. However, the difference in the AUC between the 3 algorithms was slight (0.831–0.890). Huang et al^[25,26]^ compared the performance of MLP, Gaussian Naïve Bayes, Linear Discriminant Analysis, SVM, LR, and RF algorithms for predicting molecular subtypes of BC. The results show that the MLP and LR models achieved AUCs of 0.896 and 0.881, respectively. There was no significant difference in the AUC values between the 2 models (*P* = .119). The MLP model also outperformed the other models in classifying HER2+ and HER2−, with an AUC of 0.840 and an accuracy of 79.0%.

However, existing models do not consider the HER2-low category, which is representative of most BC. In our study, we utilized 6 ML algorithms to develop these models and compared their diagnostic efficacies. Our findings revealed that all 6 algorithms exhibited high diagnostic efficacy for distinguishing between HER2 low expression and HER2-positive BC. The AUC value of all 6 algorithms was 1.000 for the training set. In the validation set, the AE models achieved an AUC value of 0.994, whereas the remaining models achieved an AUC value of 1.000. The differences in the AUC values between the models in the training and validation sets were not statistically significant. These results suggest that the ML model based on DCE-MRI radiomics is highly effective for predicting HER2 low expression and HER2-positive BC. It can provide valuable complementary information to assist in clinical decision-making and precision therapy.

### 4.1. Limitations and future recommendations

The present study has several limitations. First, this was a retrospective study, which may have introduced a selection bias. Second, although the current study showed an optimal AUC through internal validation, external validation was necessary to confirm these findings. Third, this study focused on exploring the predictive value of radiomic features; however, the clinical features, traditional imaging features, and laboratory indicators were not included. Future studies should consider combining clinical features and traditional imaging features. Finally, the radiomic features and predictive models developed in this study are currently only applicable to scientific research, and their translation into clinical practice requires further validation.

## 5. Conclusion

Our study investigated the feasibility of using DCE-MRI-based radiomics features to predict HER2-low BC. Certain radiomics features showed associations with HER2-low BC and may have predictive value. ML prediction models developed using these radiomics features could be beneficial for distinguishing between HER2-low and HER2-positive BC. These noninvasive preoperative models have the potential to assist in clinical decision-making for HER2-low breast cancer, thereby advancing personalized clinical precision.

## Acknowledgments

All authors thank the radiologists and patients at the Guangxi Medical University Cancer Hospital. We thank Paperpal (https://preflight.paperpal.com) for English language review of this manuscript.

## Author contributions

**Conceptualization:** Xianfei Chen.

**Data curation:** Xianfei Chen.

**Formal analysis:** Danke Su.

**Funding acquisition:** Xianfei Chen.

**Investigation:** Xianfei Chen.

**Methodology:** Xianfei Chen, Minghao Li.

**Project administration:** Danke Su.

**Resources:** Danke Su.

**Software:** Xianfei Chen, Minghao Li.

**Validation:** Xianfei Chen, Minghao Li.

**Visualization:** Xianfei Chen, Minghao Li.

**Writing – original draft:** Xianfei Chen.

**Writing – review & editing:** Danke Su.

## Supplementary Material


