# Supplementary material for: Machine learning models for differential diagnosing HER2-low breast cancer: A radiomics approach
Source: Medicine (Baltimore). 2024 Aug 16;103(33):e39343. doi: 10.1097/MD.0000000000039343 (PMC11332746; doi:10.1097/MD.0000000000039343)
Supplement: Supplementary file 1 [file medi-103-e39343-s001.docx]

**Supplementary Materials**

**Part 1: Feature Selection Methods**

1. **PCC (Pearson correlation coefficient)**

The PCC is a widely used filter method that ranks and selects features based on statistical correlations. Filter-based methods use some metrics to determine how independently predictive a given feature is, and those features that are most predictive while being independent of others are selected. Unlike other feature selection methods (wrapper and embedded), filter methods are independent and separate from classifier algorithms. This separation means that the filter methods are free from classifier bias, which reduces overfitting. The main advantage of filter methods over other feature selection methods is that they are generally less computationally demanding and, thus, can easily be scaled to high-dimensional data.

**(2)RFE (recursive feature elimination)**

Given an external estimator that assigns weights to features (e.g., the coefficients of a linear model), recursive feature elimination (RFE) selects features by recursively considering smaller sets of features. First, the estimator is trained on the initial set of features, and the importance of each feature is obtained either through any specific attribute or is callable. The least important features were then pruned from the current set of features. This procedure is recursively repeated on the pruned set until the desired number of features to be selected is eventually reached.

**Part 2: Machine Learning Algorithms**

1. **An auto-encoder (AE)**, also known as a Multilayer Perceptron **(MLP),**  is a supervised learning algorithm that learns a function by training on a dataset. Given a set of features and target, it can learn a nonlinear function approximator for either classification or regression. This is different from logistic regression in that there can be one or more nonlinear layers, called hidden layers, between the input and output layers. An MLP with hidden layers has a nonconvex loss function, where there exists more than one local minimum. Therefore different random weight initializations can lead to different validation accuracies. “hidden_layer_sizes”, “activation” and “solver” were used in the tuning step of model development. more details can be found at https://scikit-learn.org/dev/modules/generated/sklearn.neural_network.MLPClassifier.html

**(2) Random Forest(RF):** A random forest is a meta-estimator that fits several decision tree classifiers on various sub-samples of the dataset and uses averaging to improve the predictive accuracy and control overfitting. Trees in the forest use the best-split strategy, which is equivalent to passing splitter="best" to the underlying Decision Tree Regressor. The sub-sample size is controlled with the max_samples parameter if bootstrap=True (default), otherwise the whole dataset is used to build each tree. more details can be found at <https://scikit-learn.org/stable/modules/generated/sklearn.ensemble.RandomForestClassifier.html>

**(3) Logistic Regression(LR):** Logistic regression is a well-established technique that, despite its name, is used more generally as a classifier. Logistic regression models have a fixed number of parameters, which depend on the number of input features and output categorical predictions. This is similar to linear regression, where several points are fitted to a line, minimizing a function, such as the mean squared error (MSE). Logistic regression instead fits the data to a sigmoid function from 0 to 1, and when the output is less than 0.5, the example is assigned to one class; otherwise, it is the other. More details can be found at <http://scikit-learn.org/stable/modules/generated/sklearn.linear_model.LogisticRegression.html>

**(4)Support Vector Machine(SVM):** SVMs are based on the idea of finding a hyperplane that best divides the set of training examples into two classes. Support vectors are the examples nearest to the hyperplane, and the points of a dataset that, if removed, would alter the position of the dividing hyperplane. A hyperplane is a line that separates and classifies a set of data linearly. The goal is to determine the formula for a plane that best separates examples. This is called a hyperplane because the dimensionality of the plane is the dimension of the examples (and remembering each example is a vector of features). It is common to remap points from a simple n-dimensional space to a different type of space if it can produce a better separation of points. There are also hyperparameters (a variable that is external to the model and whose values cannot be estimated from data) that impact the development of a model. For instance, in the case of SVMs, a penalty must be assigned to an example that is on the wrong side of the decision plane. The hyperparameter is the weighting of that penalty: the weighting of no examples really wrong (therefore, assigning a high power to the error) versus fewer examples wrong, even if they are really wrong. More details can be found at https://scikit-learn.org/stable/modules/svm.html

**(5) AdaBoost (AB):** The core principle of AdaBoost is to fit a sequence of weak learners (i.e., models that are only slightly better than random guessing, such as small decision trees) on repeatedly modified versions of the data. The predictions from all of them were then combined through a weighted majority vote (or sum) to produce the final prediction. The data modifications at each so-called boosting iteration consist of applying weights $w_{1}$, $w_{2}$, …, $w_{N}$ to each of the training samples. Initially, these weights are all set such that the first step simply trains a weak learner on the original data. For each successive iteration, the sample weights were individually modified and the learning algorithm was reapplied to the reweighted data. At a given step, the training examples that were incorrectly predicted by the boosted model induced in the previous step had their weights increased, whereas the weights were decreased for those that were predicted correctly. As iterations proceed, examples that are difficult to predict receive ever-increasing influence. Each subsequent weak learner is thereby forced to concentrate on the examples that were missed by the previous ones in the sequence. More details can be found at <http://scikit-learn.org/stable/modules/generated/sklearn.ensemble.AdaBoostClassifier.html>

https://scikit-learn.org/stable/modules/ensemble.html#adaboost

**(6)Decision tree(DT):** Decision Trees are a nonparametric supervised learning method used for classification and regression. The goal is to create a model that predicts the value of a target variable by learning simple decision rules inferred from data features. A tree can be regarded as a piecewise constant approximation. More details can be found at <https://scikit-learn.org/stable/modules/tree.html>
